# CORRECTION

**DOI:** 10.1111/cas.15219

**Published:** 2022-01-10

In an article^1^ titled “VEGFR2 blockade augments the effects of tyrosine kinase inhibitors by inhibiting angiogenesis and oncogenic signaling in oncogene‐driven non‐small‐cell lung cancers” by Hiromi Watanabe, Eiki Ichihara, Hiroe Kayatani, Go Makimoto, Kiichiro Ninomiya, Kazuya Nishii, Hisao Higo, Chihiro Ando, Sachi Okawa, Takamasa Nakasuka, Hirohisa Kano, Naofumi Hara, Atsuko Hirabae, Yuka Kato, Takashi Ninomiya, Toshio Kubo, Kammei Rai, Kadoaki Ohashi, Katsuyuki Hotta, Masahiro Tabata, Yoshinobu Maeda, and Katsuyuki Kiura, the authors would like to correct the first image from the right at the top row of Figure 6B. The correct figure 6B is shown below.

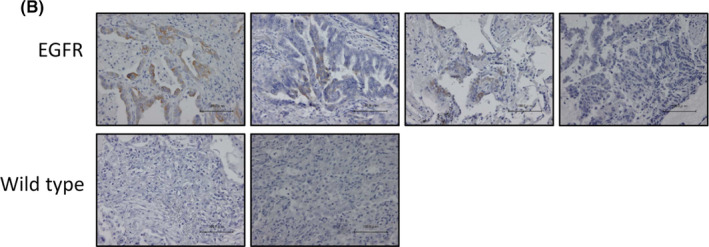

The authors apologize for the error.
